# Engineering Properties and Optimal Conditions of Cementless Grouting Materials

**DOI:** 10.3390/ma12193059

**Published:** 2019-09-20

**Authors:** Jaehyun Lee, Gyuyong Kim, Yongro Kim, Kyungju Mun, Jeongsoo Nam

**Affiliations:** 1 Department of Architectural Engineering, Chungnam National University, Yuseong-Gu, Daejeon 34134, Korea; archi0528@daum.net (J.L.); j.nam@cnu.ac.kr (J.N.); 2 Technology Research and Development Institute, Daelim Industrial, Jongno-Gu, Seoul 03152, Korea; archi0528@hotmail.com; 3 Technology Research Institute, Zian Industrial, Wanju-Gun, Jeollabuk-do 55338, Korea; mun7890@hanmail.net

**Keywords:** ordinary Portland cement, ground granulated blast-furnace slag, cementless grouting materials, soil grouting, gel time, homogel strength

## Abstract

This study aims to analyze the engineering properties of cementless grouting materials (CGMs) and derive optimal binder types and compositions that can ensure superior material performance in comparison with ordinary Portland cement (OPC). The presented CGM is an environment-friendly inorganic binder based on ground granulated blast-furnace slag. The material properties of three CGM types with different chemical compositions were evaluated. To assess the possibility of using CGMs in grouting-construction methods, this study followed special grouting-method specifications of the J company in Korea, and tested whether CGM satisfies the performance requirements of a gel time of 20–50 s and homogel strength greater than 2 MPa after 7 days. For OPC and CGM, gel time increased and homogel strength decreased as the water/binder (W/B) ratio of Liquid B increased or as its replacement ratio decreased. Additionally, gel time decreased while homogel strength increased as the absolute weight of the Liquid B binder increased, and a negative correlation was observed between gel time and homogel strength. CGM2 was the optimal binder to ensure excellent material performance compared with OPC. Optimal mixing proportions were 117.8–167.7% W/B ratio, 42.6–56.7% Liquid B volume ratio, and 20.4–43.7 kg binder weight.

## 1. Introduction

Soil-improvement methods were first developed in England in 1962 with jet-grouting methods to improve retention walls [1]. Currently, there are a number of studies on the development of these methods to resolve various geological engineering issues [2]. The injection and grouting method is a typical soil-improvement method based on injecting a chemical liquid grouting material (CLGM) into the soil to improve it according to certain performance requirements. This method is widely used for earth-retention purposes and, occasionally, for the purpose of soil hardening. Among the materials that compose CLGM, sodium silicate number 3 (SS No. 3) is typically used for “Liquid A,” which is the main ingredient responsible for setting, and ordinary Portland cement (OPC) is used for “Liquid B,” which acts as the binder.

SS (No. 3) has been developed for application in various grouting methods and is commonly used as a solution injecting material [3]. SS (No. 3) is composed of sodium silicate and calcium chloride, which acts as an activator and can react with OPC, yielding strong adhesive properties. A mixture of these two materials has been used for geotechnical grouting with significant amounts of moisture and water. Furthermore, this mixture can form a permanent wall that can secure the required strength even when the material is not dry [4,5,6].

However, there is a concern that, when soil-grouting material consisting of SS (No. 3) is injected into the ground, the wall-permeability effect may not be observed owing to the alkali-leaching phenomenon and shrinkage of the hardened material.

When OPC is manufactured, a large amount of CO_2_ is generated, accounting for approximately 7% of anthropogenic CO_2_ emissions; hence, substitute materials for conventional CLGM Liquid B are being actively developed to reduce CO_2_ generation, not only for performance purposes but also for environmental reasons [7,8]. 

A method that reduces CO_2_ generation, or at least prevents it from increasing, is one that replaces OPC with an industrial by-product, blast-furnace slag (BFS), as a binder, or one that uses cementitious materials that guarantees improved environmental and technical performance [9,10]. 

In particular, alkali-based materials, which are cementitious materials, exhibit excellent material and environmental properties, which is why global interest has increased in these materials. 

These materials form from a mixture of a solid precursor, such as calcium silicate or aluminosilicate, and an alkali activator. Alkali hydroxides and silicates, such as NaOH or Na_2_SiO_3_, are usually used as the activator. Fly ash and BFS are among the most commonly used materials as solid precursors [11,12,13,14,15,16]. 

BFS is a by-product of pig-iron manufacturing during the steel-manufacturing process, which can yield approximately 300 kg of BFS for every ton of pig iron [17]. An alkali-activated slag-based inorganic binder exhibits high mechanical strength and excellent durability in corrosive environments [18,19,20,21,22]. 

Fly ash is a by-product of coal burning in thermal-power plants. An inorganic binder that includes alkali-activated fly ash generally possesses high strength and durability in harsh environments. Fly ash is known to have reduced shrinkage and excellent heat resistance; thus, it is widely used in modern jet-grouting methods [23,24,25,26]. 

Recently, research on alkali-based materials has been actively conducted. Alkali-based materials can be classified into two main categories depending on calcium content: low-calcium metakaolin or FA-based geopolymers, and alkali-based materials using BFS containing large amounts of calcium [27,28,29]. Alkali-based materials are superior to cement in terms of technical performance, and their main advantages are their mechanical properties, such as their strength and durability. Consideration of durability is crucial with respect to certain methods, such as soil grouting, in which materials are injected into the soil. Furthermore, OPC is chemically vulnerable to sulfates potentially found in the soil [30].

If an appropriate activator is selected to improve the reactivity of BFS and fly ash, it is possible to achieve superior properties in terms of mechanical, environmental, and economic performance in comparison with existing materials.

Circulating fluidized bed combustion (CFBC) ash is an industrial by-product of the coal-combustion process in a circulating fluidized bed boiler. The mineral and chemical compositions, as well as the shape of the CFBC ash, are different from the properties of ash produced in conventional pulverized coal combustion (PCC) [31,32,33,34]. CFBC ash is especially not suitable for concrete admixtures owing to its high ignition loss and CaO content. However, it is highly suitable for ground applications, as it has a high CaO content and can be used as an alkali activator. In addition, as petro cokes desulfurization gypsum (PCDG) is mixed with limestone during the petrocoke-combustion process, which is used as a CFBC-type boiler fuel, the CaO and SO_3_ content acts as sulfate activator because of the decarboxylation process and the desulfurization reaction of limestone. This has led to the use of industrial by-products as new soil-grouting materials that have not been analyzed in previous studies.

Therefore, based on analysis of previously performed experiments, this study focuses on determining whether it is possible to replace conventional OPC grouting with cementless grouting materials (CGMs), which are defined as alkali-based materials created through a process of mixing and pulverizing 50–60% BFS, 30–40% CFBC ash, and 0–20% PCDG.

As CFBC ash has a relatively high content of CaO, it is characterized by a self-hydraulic property and can produce Ca(OH)_2_ through hydration reactions, thereby easily securing initial strength. Furthermore, Ca(OH)_2_ not only stimulates BFS, but also reacts with silicate or aluminate contained in the ground to produce calcium silicate hydrate (C-S-H) and calcium aluminate hydrate (C-A-H). In addition, CFBC ash and the PCDG sulfate activator serve to activate the surface of the BFS particles so that they obtain hardening characteristics similar to OPC.

**Figure 1:**
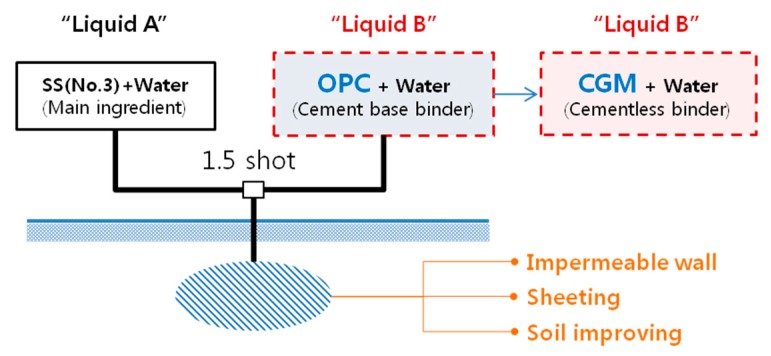
Conceptual diagram of chemical-grouting method.

Because the main ingredient, Liquid A, affects the congealing speed for gel time, and Liquid B affects the strength of the hardened material, it is important to examine the factors involved with mixing Liquids A and B to be able to use CGM as a substitute for conventional OPC (Figure 1). This study evaluates the gel time and homogel strength of OPC and CGM according to certain mixing factors, such as the type and water/binder (W/B) ratio of the liquid binder used and the replacement ratio of Liquid B. Based on the results of the performance evaluation using these factors, the optimal type and CGM composition with excellent material performance were derived and compared with OPC.

In the field of concrete and mortar, many studies on cementless applications have been conducted. These studies focus on latent hydraulic induction by adding stimulants to BFS. However, in the field of grouting, no studies have been conducted using cementless binders. In particular, the novelty of this study lies in the fact that it could derive the optimal binder type and CGM composition that could secure superior performance compared to OPC by mixing SS (No. 3) and cementless binder through various tests.

## 2. Materials and Methods

### 2.1. Materials

Table 1 lists the physical and chemical properties of SS (No. 3), which was used as Liquid A, whereas Table 2 lists the physical and chemical properties of OPC and CGM 1, 2, and 3, which were used as the Liquid B binder. The CGMs used in this study were inorganic binders based on BFS, manufactured using fluidized bed combustion fly ash and an alkali/sulfate activator. This process enabled the reactivity of the amorphous material, induces hydrate formation, and developed strength in the absence of cement [35]. CGM 1, 2, and 3 have different chemical compositions, whose densities were 2.82, 2.89, and 2.70 g/cm^3^, respectively, equating to 86–92% of OPC density. On the other hand, the OPC, CGM 1, 2, and 3 powders were 3120, 4223, 4190, and 4172 cm^2^/g, respectively.

Table 3 lists the chemical composition of CGM. Furthermore, the mass ratio of BFS:CFBCash:PCDG for CGM 1, 2, and 3 was 50%:40%:10%, 50%:30%:20%, and 60%:40%:0%, respectively.

### 2.2. Experiment Plan

Table 4 summarizes the experiment plan of this study. Water temperature was maintained constant at 20 °C. In addition, 4 types of Liquid B binders were used: conventionally used binder OPC and BFS-based CGM 1, 2, and 3 binders. In general, major factors that determine the physical and mechanical properties of a retention wall are the W/B ratio and replacement ratio of Liquid B. Previous studies typically used a W/B ratio of 100% and a Liquid B replacement ratio of 50% [36,37,38]. In this study, to examine a variety of mixing factors, the W/B ratio of Liquid B varied at 100%, 120%, and 140%, and the volume replacement ratio of Liquid B varied at 50%, 60%, and 70%. For the evaluation criteria, the major material properties, i.e., gel time and homogel strength, were measured at 7 days to aid in the evaluation of the possibility of using a soil-grouting CLGM. All experiments were performed in an environment with an air temperature of 20 ± 2 °C and humidity of 30–40% RH, as recommended by the National Conference of Standards Laboratories–International (NCSLI). Table 5 lists the CLGM mixtures used in this study.

### 2.3. Test Methods

#### 2.3.1. Gel Time

Gel time is an important material property of grouting CLGMs. If Liquids A and B are mixed, the viscosity of the mixed liquid gradually increases until it loses its liquidity and gelling occurs. Gel time is the amount of time that elapses between mixing the grouting CLGM and loss of liquidity/beginning of gelling. 

**Figure 2:**
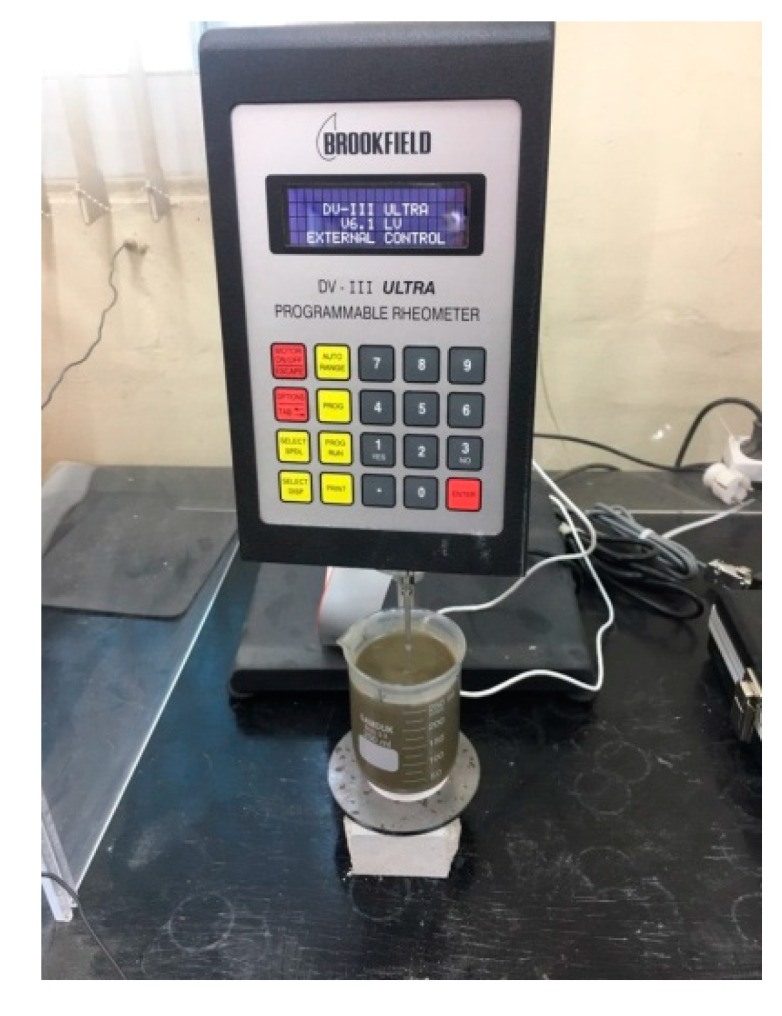
Test setup to measure gel time.

To measure gel time, we selected the viscometer method, whose test setup (Brookfield digital rheometer dv-III, Brookfield Engineering Laboratories, INC., MA, USA) is illustrated in Figure 2. The gel time of Liquids A and B was measured according to each mixing factor. The viscometer began rotating at 10 s after mixing, and the required time to reach a viscosity of 100 cP was measured. Gel time was measured thrice for each mixing condition, and average gel time was calculated. Following the special grouting-method specifications from the J company [39], target gel time was set between 20 and 50 s.

#### 2.3.2. Homogel Strength

Homogel strength refers to the compressive strength of the solidified material that only hardens the injection material, whereas sandgel strength refers to the compressive strength of the solidified material that penetrates the sand and hardens the injection material. In this study, homogel strength was tested.

**Figure 3:**
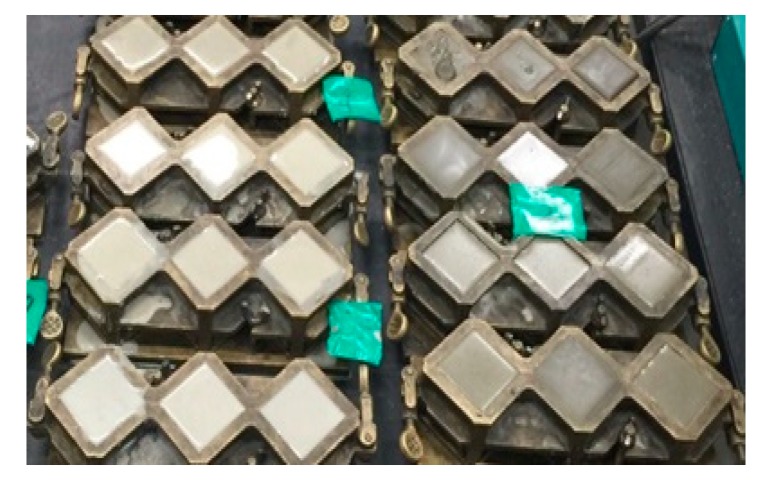
Specimens for homogel compressive-strength test.

As depicted in Figure 3, 50 mm × 50 mm × 50 mm cubic specimens were cast according to ASTM C109/C109M-16a (Standard Test Method for Compressive Strength of Hydraulic Cement Mortars) [40], and water curing was performed at a temperature of 20 ± 2 ℃. Then, uniaxial compressive strength was measured after 3 and 7 days for each mixing condition for 3 specimens, and the average of these values was reported. Measurements were taken after 7 days to monitor the development of compressive strength. In general, at a construction site, a target is expected to have a homogel strength of 2 MPa or more after 7 days.

## 3. Results and Discussion

### 3.1. Material Properties According to Liquid B W/B ratio

#### 3.1.1. Gel-Time Properties According to W/B Ratio by Binder Type

**Figure 4:**
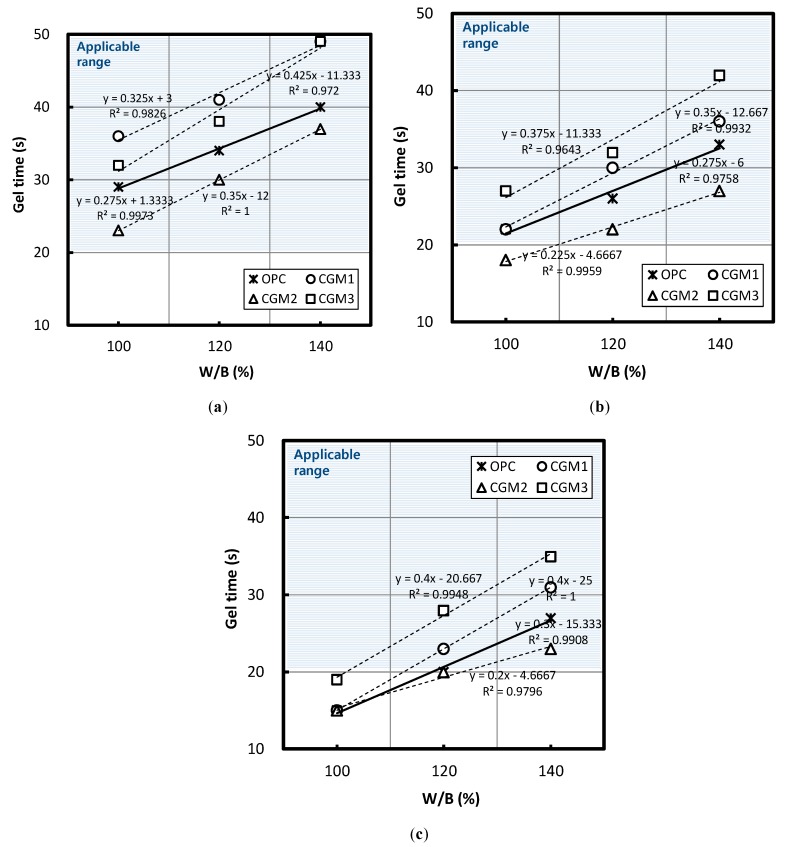
Gel time based on water/binder (W/B) ratio of Liquid B for Liquid B/CLGM equal to (**a**) 50%, (**b**) 60%, and (**c**) 70%.

Figure 4 illustrates the changes in gel time according to the W/B ratio when the replacement ratio of Liquid B was 50%, 60%, and 70%. Regardless of binder type, when the W/B ratio of Liquid B increased, gel time also increased. According to a regression equation based on the experiment results, when the W/B ratio of Liquid B increased by 10%, gel times of CGM 1, 2, 3, and OPC increased by a minimum of 2 and maximum of 4.25 s, where the average values for each gel time were 3.58, 2.58, 4, and 2.83 s, respectively. The ratio of increase in gel time for CGM 1 and 3 compared with OPC was 26.5% and 41.3%, respectively; however, the ratio for CGM 2 was −8.8%. In addition, considering that gel-time target values were 20–50 s, none of the specimens with a Liquid B replacement ratio of 70% and a W/B ratio of 100% was within the desired range, but all were classified as quick-setting, and this must be taken into account when setting the mixing factors for water glass grouting methods. The average absolute values for each gel time with exposure to identical mixing conditions were ranked in the following order: CGM 1 > CGM 3 > OPC > CGM 2. The chemical composition of CGM 2 was found to be the most effective composition to reduce gel time when exposed to identical mixing conditions.

Following a regression equation based on the experiment results, Table 6 lists the estimated values for the applicable range of W/B ratio of Liquid B that satisfied the target gel time, as well as the estimated values for W/B ratio and GCM gel time ranges that could reduce gel times in comparison with the OPC. Through exposure to identical mixing conditions, CGM 2 was characterized by improved performance with respect to reducing gel time compared with the OPC in all ranges that satisfied the target gel time when the volume replacement ratio of Liquid B was 50%, 60%, and 70%. In addition, it was impossible to reduce gel time by using CGM 1 and 3 in the applicable range of the W/B ratio, regardless of volume ratio of Liquid B, whereas CGM 2 was capable of reducing gel time by 117.8–177.1%.

#### 3.1.2. Homogel Strength According to W/B Ratio by Binder Type

**Figure 5:**
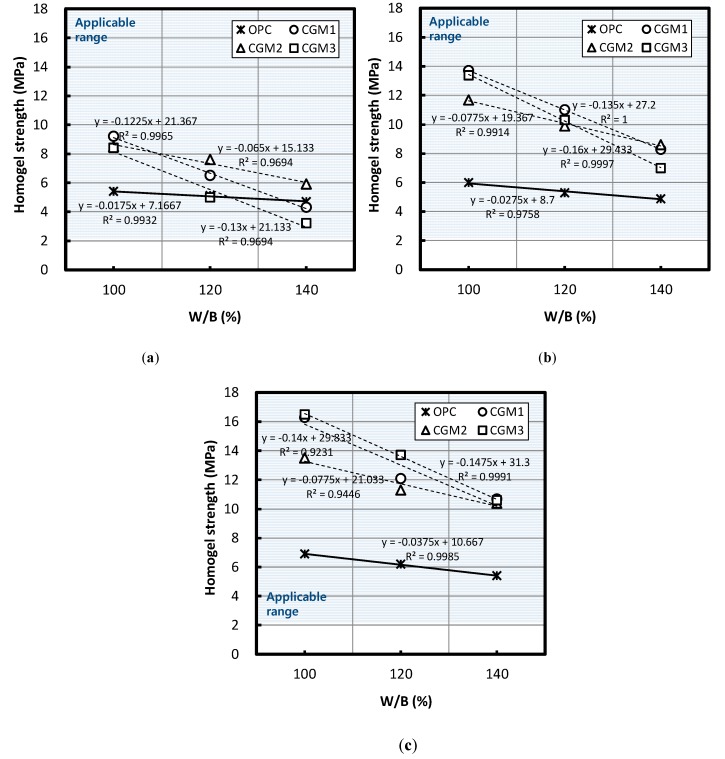
Homogel strength based on W/B ratio of Liquid B for a Liquid B/CLGM equal to (**a**) 50%, (**b**) 60%, and (**c**) 70%.

Figure 5 illustrates the changes in homogel strength according to the W/B ratio of Liquid B and age when the volume replacement ratio of Liquid B was 50%, 60%, and 70%. Figure 5 illustrates that, regardless of binder type, homogel strength decreased as W/B ratio increased.

According to a regression equation based on the experiment results, when the W/B ratio increased by 10%, the homogel strength of CGM 1, 2, 3, and OPC after seven days resulted in the following, ranked in descending order: CGM 3 (1.30 MPa) > CGM 1 (1.23 MPa) > CGM 2 (0.65 MPa) > OPC (0.18 MPa), CGM 3 (1.60 MPa) > CGM 1 (1.35 MPa) > CGM 2 (0.78 MPa) > OPC (0.28 MPa), CGM 3 (1.48 MPa) > CGM 1 (1.40 MPa) > CGM 2 (0.78 MPa) > OPC (0.38 MPa). Each of the average values ranked in the following order: CGM 3 (1.46 MPa) > CGM 1 (1.33 MPa) > CGM 2 (0.74 MPa) > OPC (0.28 MPa). The increase in ratios for homogel strength of CGM 1, 2, and 3 compared with OPC was 474%, 263%, and 521%, respectively. Regardless of type, the range of reduction in the homogel strength of CGM with the increase in W/B ratio was large compared with that of OPC.

Moreover, homogel strength after seven days exceeded 2 MPa for all replacement ratios of Liquid B used in this study (i.e., 50%, 60%, and 70%) and W/B ratios (i.e., 100%, 120%, and 140%); hence, it was within the range of applicability. 

Under identical mixing conditions, the absolute values of homogel strength after seven days were higher in all CGM specimens compared with that of OPC with the exception of the CGM 3 (Liquid B replacement ratio of 50% and W/B ratio of 140%, Liquid B replacement ratio of 50% and W/B ratio of 120%) and CGM 1 (Liquid B replacement ratio of 50% and W/B ratio of 140%) specimens. These results indicate that BFS-based CGMs can guarantee effective homogel strength if used at an actual site.

Based on a regression equation fitted to the experiment results, Table 7 lists the estimated values of the applicability range for all binder types that satisfied the target homogel strength, in addition to listing the estimated values of points that had greater homogel strength than that of OPC. Between the CGM 1, 2, and 3 specimens, CGM 2 was effective at increasing homogel strength within the widest range of Liquid B replacement ratios. 

Based on an examination of the applicability range of the W/B ratio of Liquid B that could improve homogel strength in comparison with that of OPC, we observed that CGM 1 improved homogel strength at 135.2% or less, CGM 2 at 167.7% or less, and CGM 3 at 124.1% or less.

### 3.2. Material Properties According to Volume Replacement Ratio of Liquid B

#### 3.2.1. Gel-Time Properties According to Liquid B Replacement Ratio by Binder Type

**Figure 6:**
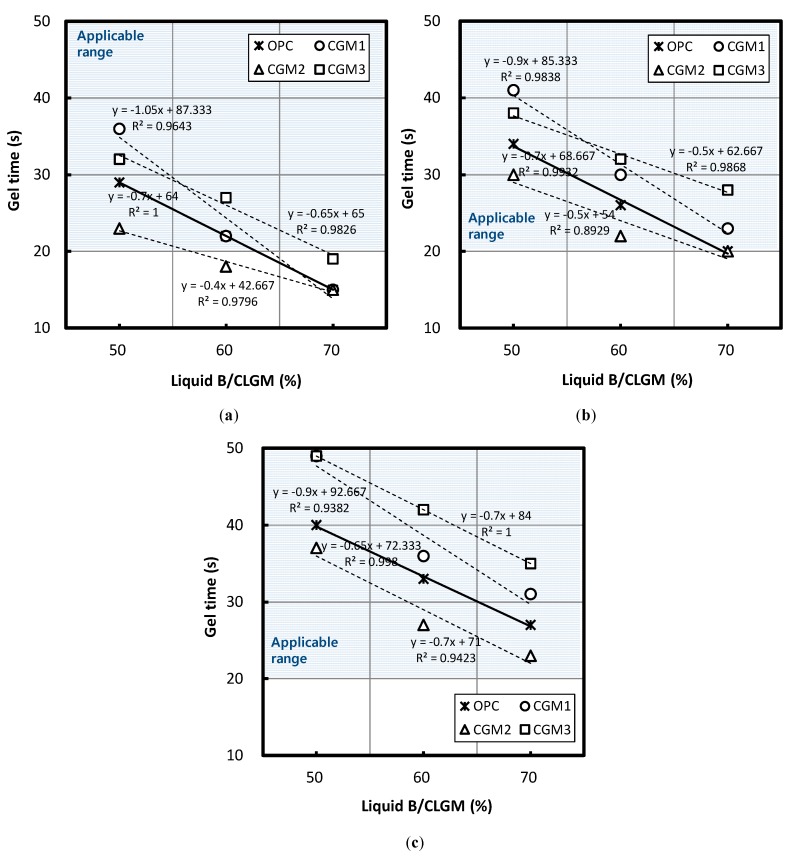
Gel time based on volume replacement ratio of Liquid B for a W/B ratio equal to (**a**) 100%, (**b**) 120%, and (**c**) 140%.

Figure 6 illustrates the changes in gel time according to the Liquid B replacement ratio when the W/B ratio of Liquid B was 100%, 120%, and 140%. Figure 6 depicts that there was a reduction in gel time as the Liquid B replacement ratio increased, regardless of binder type.

When the volume ratio of Liquid B increased by 10%, gel time for CGM 1, 2, 3, and OPC increased by a minimum of 4 and a maximum of 10.5 s, with the average values for each gel time being 9.5, 5.33, 6.17, and 6.83 s, respectively. The gel-time reduction of CGM1 was 39.1% greater than that of OPC; however, CGM 2 and 3 exhibited a reduction of 22% and 9.7%, respectively.

Furthermore, considering that gel-time target values were 20–50 s, none of the specimens with a Liquid B replacement ratio of 70% and a W/B ratio of 100% was within the applicable range, and they were all classified as quick-setting. Furthermore, at a Liquid B replacement ratio of 60%, the gel time of CGM 2 was under 20 s, which must be taken into account when setting the mixing factors for water glass grouting methods. 

The average absolute values for gel times with exposure to identical mixing conditions ranked in the following order: CGM 1 > CGM 3 > OPC > CGM 2. The chemical composition of CGM 2 was the most effective at reducing gel time under identical mixing conditions.

Following a regression equation based on the experiment results, Table 8 presents the estimated values for the applicable range of the replacement ratio of Liquid B that satisfied the target gel time, in addition to listing the estimated values for the replacement ratio and gel-time ranges of CGMs that could reduce gel times in comparison with OPC. When exposed to identical mixing conditions, CGM 2 exhibited improved performance with respect to reducing gel time in comparison with OPC in all ranges that satisfied the target gel time when the W/B ratio was 100%, 120%, and 140%. In addition, CGM 1 and 2 could not be applied to the range of volume replacement ratios of Liquid B that could reduce gel time relative to that of OPC, regardless of the W/B ratio of Liquid B. However, CGM 2 could reduce gel time by 30.0–56.7%.

#### 3.2.2. Homogel Strength According to Volume Replacement Ratio of Liquid B by Binder Type

**Figure 7:**
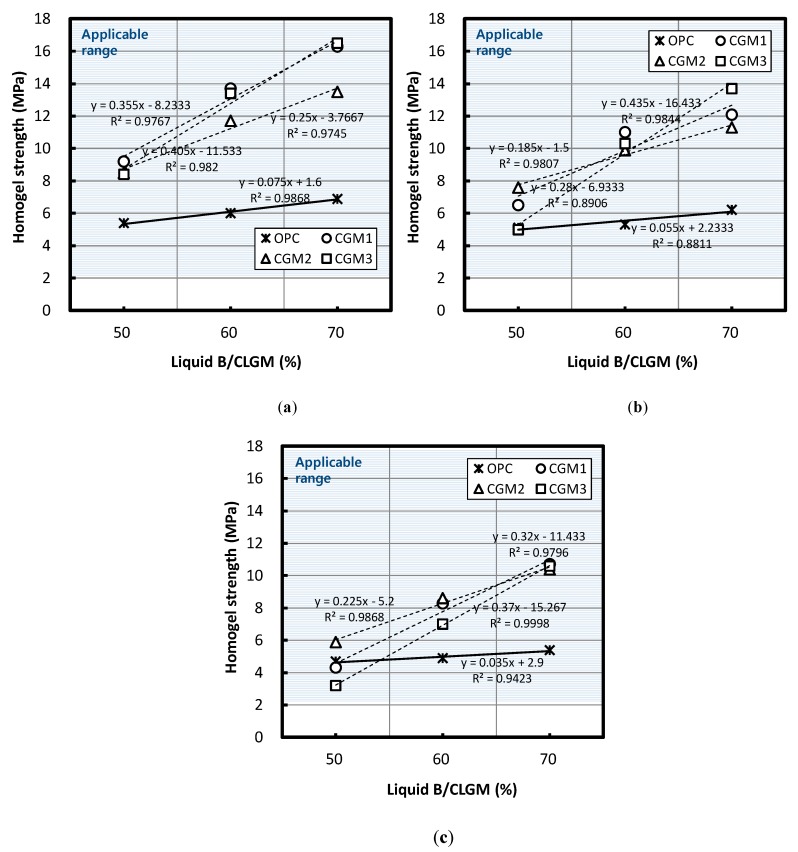
Homogel strength based on volume replacement ratio of Liquid B for a W/B ratio equal to (**a**) 100%, (**b**) 120%, and (**c**) 140%.

Figure 7 depicts changes in homogel strength according to the volume replacement ratio of Liquid B when the W/B ratio was 100%, 120%, and 140%. Figure 7 shows that, regardless of binder type, homogel strength increased with an increase in the replacement ratio of Liquid B.

According to a regression equation based on the experiment results, when the replacement ratio of Liquid B increased by 10%, the homogel strength of CGM 1, 2, 3, and OPC after seven days ranked in the following order: CGM 3 (4.05 MPa) > CGM 1 (3.55 MPa) > CGM 2 (2.50 MPa) > OPC (0.75 MPa), CGM 3 (4.35 MPa) > CGM 1 (2.80 MPa) > CGM 2 (1.85 MPa) > OPC (0.55 MPa), CGM 3 (3.70 MPa) > CGM 1 (3.20 MPa) > CGM 2 (2.25 MPa) > OPC (0.35 MPa). Each of the average values ranked in the following order: CGM 3 (4.03 MPa) > CGM 1 (3.18 MPa) > CGM 2 (2.20 MPa) > OPC (0.55 MPa). The decrease in ratios of homogel strength of CGM 1, 2, and 3 compared with OPC was 579%, 400%, and 733%, respectively. Regardless of type, the range in reduction of CGM homogel strength was larger than that of OPC when the Liquid B replacement ratio increased.

On the other hand, the homogel strength of OPC, CGM 1, 2, and 3 was 5.0, 9.1, 8.7, and 8.6 MPa, respectively, indicating that the homogel strength of CGM was 171.9–182.2% higher than that of OPC. CGM fines exhibited a high value ranging from 133.7–135.4% in comparison with that of OPC, which could enhance homogel strength.

According to a regression equation based on the experiment results, Table 9 lists the estimated values of the applicability range by binder type that satisfied the target homogel strength, in addition to listing the estimated values of the points that had greater homogel strength than that of OPC. From the CGM 1, 2, and 3 specimens, CGM 2 was effective at increasing homogel strength within the widest range of Liquid B replacement ratios. 

Based on an examination of the applicability range of the volume ratio of Liquid B that could improve homogel strength relative to that of OPC, we observed that homogel strength could be increased by 50.3–100.0% for CGM 1, 42.6–100.0% for CGM 2, and 54.2–100.0% for CGM 3.

### 3.3. Material Properties According to Binder Weight

In this study, experiments were performed by setting the mixing factors (i.e., the W/B and volume replacement ratio of Liquid B) to three different levels for each binder type. To ensure rapid and easy mixing, as well as efficient quality management for each binder type at an actual site, the decision was made to combine the two variables, i.e., the W/B and replacement ratio of Liquid B, with the single variable, i.e., the binder content of Liquid B, such that additional observations could be made regarding changes in gel time and homogel strength.

**Figure 8:**
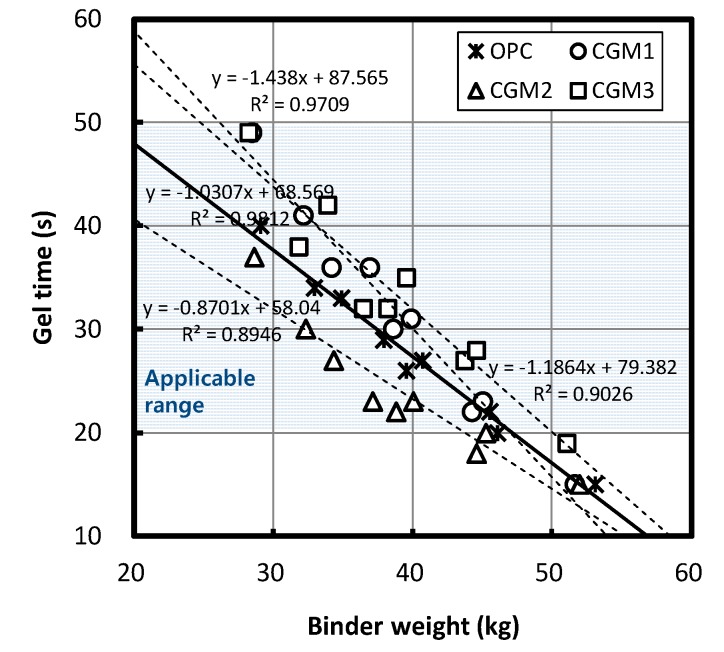
Gel time by binder weight.

Figure 8 depicts changes in gel time according to the Liquid B binder content by each binder type, where gel time appeared to decrease as the Liquid B binder content increased, regardless of binder type. According to a regression equation based on the experiment results, when the binder content increased by 10 kg, the gel times for CGM 1, 2, 3, and OPC decreased by 14.38, 8.7, 11.86, and 10.31 s, respectively, and from CGM 1, 2, and 3, only the gel-time reduction ratio of CGM2 was slightly lower than that of OPC. 

**Figure 9:**
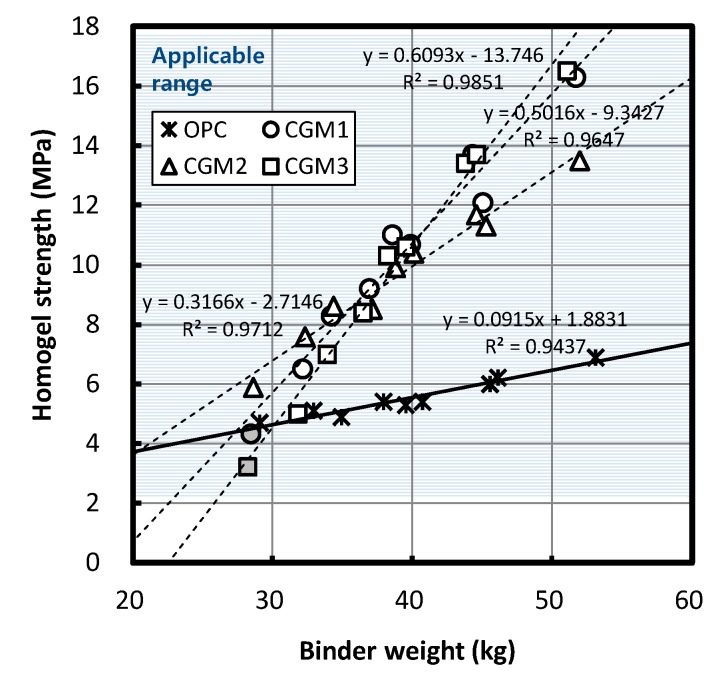
Homogel strength by binder weight.

Figure 9 depicts changes in homogel strength after three and seven days according to the Liquid B binder content. Homogel strength appeared to increase as the Liquid B binder content increased, regardless of binder type. According to the regression equation, when the binder content increased by 10 kg, the homogel strength of CMG 1, 2, 3, and OPC after seven days increased by 5.02, 3.17, 6.10, and 0.92 MPa, respectively. Therefore, the ratios of the increase in homogel strength for CGM 1, 2, and 3 were 546%, 345%, and 663% relative to OPC, respectively.

### 3.4. Correlations between Homogel Strength and Gel Time

**Figure 10:**
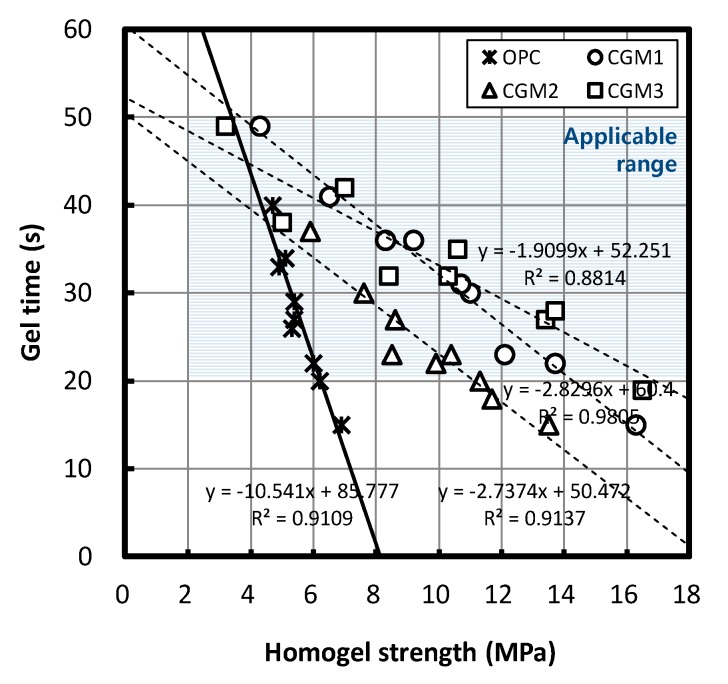
Correlations between homogel strength and gel time.

Figure 10 illustrates the correlation between homogel strength and gel time for both the CGMs and OPC. A negative correlation between homogel strength and gel time could be observed for both the CGMs and OPC. According to the regression equation, when homogel strength after seven days increased by 1 MPa, the CGM 1, 2, 3, and OPC gel times decreased by 2.83, 2.74, 1.91, and 10.54 s, respectively, and the OPC seven-day gel time changed by 454% relative to CGMs. If we targeted a higher homogel strength with identical materials and mixing conditions when using CGM 1, 2, 3, and OPC at a site, gel time could be reduced, and mixing conditions could be appropriately changed by considering the regression equations that were derived for each material in this study. 

Furthermore, within the range of gel times below 43.2 s, the homogel strength of the CGMs was greater than that for OPC at an identical level of gel time. At a homogel-strength range above 4.04 MPa, the gel time of CGM was lower than that of OPC. All specimens that were manufactured according to the mixing experiments developed in this study exceeded the target homogel strength of 2 MPa; however, five specimens (four CGM and one OPC specimen) were not within the target gel time of 20–50 s.

On the other hand, we observed that the applicability range of binder weight that could reduce gel time relative to that of OPC was impossible in CGM 1 and 3, whereas CGM 2 could reduce gel time by 9.2–43.7%. Furthermore, the applicability range of binder weight that could improve homogel strength relative to that of OPC was 27.4 kg or more for CGM 1, 20.4 kg or more for CGM 2, and 30.2 kg or more for CGM 3.

### 3.5. Derivation of Optimal Binder Type and Mixture Proportions

The results summarized in Table 6, Table 7, Table 8 and Table 9 and Figure 10 indicate that CGM 2 was the optimal binder material that ensured excellent material performance in comparison with OPC. In addition, the optimal mixture proportions of CGM 2 were a W/B ratio of 117.8–167.7%, Liquid B volume of 42.6–56.7%, and binder weight of 20.4–43.7 kg.

## 4. Conclusions

In this study, the material properties of CGMs, which could replace conventional OPC as soil-grouting material, were examined in terms of mixing conditions by comparing and observing gel time and homogel strength according to Liquid B binder type, W/B ratio, and replacement ratio. We obtained the following conclusions:

1. As the W/B ratio of Liquid B increased or its replacement ratio decreased, gel time increased while homogel strength decreased. Under identical mixing conditions, the gel time of CGM 2 was lower than that of OPC. Therefore, it is plausible that the use of CGM2 may be advantageous in situations that require rapid setting. Furthermore, under identical mixing conditions, the homogel strength of CGM 1, 2, and 3 tended to be higher than that of OPC; hence, CGM can be considered superior to OPC in terms of homogel strength.
2. As the absolute weight of the Liquid B binder increased, gel time decreased and homogel strength increased. Thus, we confirmed that the absolute weight of the Liquid B binder had an effect on gel time and homogel strength. To ensure rapid and easy mixing, and efficient quality management for each Liquid B binder type at an actual site, we suggest a combination of the two variables, i.e., W/B and volume replacement ratios of Liquid B, with the single variable, i.e., binder content of Liquid B.
3. Regardless of binder type and age, gel time and homogel strength exhibited negative correlation. If higher homogel strength were targeted with identical materials and mixing conditions when using CGM 1, 2, 3 and OPC at a site, gel time could be reduced and mixing conditions could be appropriately changed by considering the regression equations that were derived for each material in this study.
4. In this study, we determined that the liquid chemical grouting materials for soil grouting that use CGMs have material properties that satisfy special grouting method specifications, for which CGMs can be used as a substitute for conventional OPC for soil grouting.
5. In CGM 2, when the W/B ratio of Liquid B was 100%, 120%, and 140% and the Liquid B replacement ratio was in the range of 30.7–56.7%, 28.7–68.0%, and 42.6–72.9%, or when the Liquid B replacement ratio was 50%, 60%, and 70% and the W/B ratio was in the range of 91.4–167.7%, 109.6–213.3%, and 117.8–217.8%, it was possible to ensure shorter gel time and higher homogel strength in comparison with those for conventional OPC.
6. We observed that CGM 2 was the optimal binder type to improve homogel strength and reduce gel time relative to those properties for OPC at W/B ratios of 100%, 120%, and 140% of Liquid B and volume replacement ratios of 50%, 60%, and 70% of Liquid B. In addition, the optimal mixture proportions of CGM2 were a W/B ratio of 117.8–167.7%, Liquid B volume ratio of 42.6–56.7%, and binder weight of 20.4–43.7 kg.
7. In future studies, we aim to conduct microstructure analysis to further investigate the reaction mechanism of CGM 2 and determine why it exhibited superior performance when compared to OPC.

## Figures and Tables

**Table 1:** Chemical composition and physical properties of used chemical liquids. SS, sodium silicate.

Materials	Chemical Composition (%)					Density (g/cm^3^)	pH (at 25 °C)	Viscosity (at 25 °C, Pa·s)
	H_2_O	SiO_2_	Na_2_O	Fe_2_O_3_	WI ^(1)^			
SS (No. 3)	63.6	27.2	9.14	0.0034	0.0026	1.384	14	0.2

^(1)^ WI: Water insolubility.

**Table 2:** Chemical composition and physical properties of used binders. OPC, ordinary Portland cement.

Materials	Chemical Composition (%)							Density (g/cm^3^)	Fineness (cm^2^/g)
	SiO_2_	Al_2_O_3_	Fe_2_O_3_	CaO	MgO	SO_3_	Other		
OPC	17.20	4.38	3.13	66.70	3.03	3.48	2.08	3.15	3120
CGM 1	20.72	8.29	0.51	56.28	2.31	10.51	1.38	2.82	4223
CGM 2	17.60	7.01	0.52	58.85	2.02	12.73	1.27	2.89	4190
CGM 3	25.94	11.06	2.25	48.14	2.76	7.84	2.01	2.70	4172

**Table 3:** Chemical composition of raw materials that comprised cementless-grouting-material (CGM) binders. CFBC, circulating fluidized bed combustion; PCDG, petro cokes desulfurization gypsum.

Materials	Chemical Composition (%)							
	SiO_2_	Al_2_O_3_	Fe_2_O_3_	CaO	MgO	SO_3_	K_2_O	Other
BFS	32.70	13.00	0.56	45.90	4.21	1.80	0.54	1.28
CFBC ash	28.40	18.40	3.16	37.40	2.56	6.72	0.52	2.84
PCDG	1.45	0.52	0.46	72.50	1.62	22.40	0.20	0.86

**Table 4:** Experiment plan. Note: CLGM, chemical liquid grouting material.

Evaluation Items		Experiment Variables
Mixture factors	Types of liquid chemical	SS (No. 3)
	Water temperature	20 °C
	Types of Liquid B binders	OPC, CGM1, CGM2, CGM3
	W/B ratio of Liquid B	100%, 120%, 140%
	Volume replacement ratio of Liquid B (Liquid B/CLGM)	50%, 60%, 70%
Test properties	Gel state	Gel time
	Hardened state	Homogel strength (7 days)

**Table 5:** CLGM composition.

Mix No. ^(1)^	CLGM (kg)				Mix No.	CLGM (kg)			
	Liquid A		Liquid B			Liquid A		Liquid B	
	S ^(2)^	W ^(3)^	B ^(4)^	W		S	W	B	W
100-50-OPC	34.6	25.0	38.0	38.0	120-60-CGM2	27.7	20.0	38.8	46.6
100-50-CGM1	34.6	25.0	36.9	36.9	120-60-CGM3	27.7	20.0	38.2	45.8
100-50-CGM2	34.6	25.0	37.1	37.1	120-70-OPC	20.8	15.0	46.1	55.4
100-50-CGM3	34.6	25.0	36.5	36.5	120-70-CGM1	20.8	15.0	45.0	54.0
100-60-OPC	27.7	20.0	45.5	45.5	120-70-CGM2	20.8	15.0	45.3	54.3
100-60-CGM1	27.7	20.0	44.3	44.3	120-70-CGM3	20.8	15.0	44.6	53.5
100-60-CGM2	27.7	20.0	44.6	44.6	140-50-OPC	34.6	25.0	29.1	40.8
100-60-CGM3	27.7	20.0	43.8	43.8	140-50-CGM1	34.6	25.0	28.5	39.9
100-70-OPC	20.8	15.0	53.1	53.1	140-50-CGM2	34.6	25.0	28.6	40.1
100-70-CGM1	20.8	15.0	51.7	51.7	140-50-CGM3	34.6	25.0	28.2	39.5
100-70-CGM2	20.8	15.0	52.0	52.0	140-60-OPC	27.7	20.0	34.9	48.9
100-70-CGM3	20.8	15.0	51.1	51.1	140-60-CGM1	27.7	20.0	34.2	47.9
120-50-OPC	34.6	25.0	32.9	39.5	140-60-CGM2	27.7	20.0	34.4	48.1
120-50-CGM1	34.6	25.0	32.2	38.6	140-60-CGM3	27.7	20.0	33.9	47.4
120-50-CGM2	34.6	25.0	32.3	38.8	140-70-OPC	20.8	15.0	40.8	57.1
120-50-CGM3	34.6	25.0	31.8	38.2	140-70-CGM1	20.8	15.0	39.9	55.9
120-60-OPC	27.7	20.0	39.5	47.4	140-70-CGM2	20.8	15.0	40.1	56.1
120-60-CGM1	27.7	20.0	38.6	46.3	140-70-CGM3	20.8	15.0	39.5	55.4

^(1)^ W/B ratio of Liquid B—Liquid B/CLGM—types of Liquid B binders; ^(2)^ SS No. 3; ^(3)^ water; ^(4)^ binder.

**Table 6:** Estimated values of W/B ratio and gel time with volume replacement of Liquid B.

Liquid B/CLGM (Vol.%)	Item		Estimated Values			
			CGM 1	CGM 2	CGM 3	OPC
50	Applicable range of W/B ratio	Maximum limit (%)	144.6	177.1	144.3	177.0
		Minimum limit (%)	52.3	91.4	73.7	67.9
		Limit range (%)	92.3	85.7	70.6	109.1
	Range lower than OPC	W/B ratio (%)	-	91.4–177.1	73.7–84.4	-
		Gel time (s)	-	20.0–50.0	20.0–24.6	-
60	Applicable range of W/B ratio	Maximum limit (%)	179.0	243.0	163.6	203.6
		Minimum limit (%)	93.3	109.6	83.6	94.5
		Limit range (%)	85.7	133.3	80.0	109.1
	Range lower than OPC	W/B ratio (%)	-	109.6–243.0	-	-
		Gel time (s)	-	20.0–50.0	-	-
70	Applicable range of W/B ratio	Maximum limit (%)	187.5	217.8	176.7	217.8
		Minimum limit (%)	112.5	117.8	101.7	117.8
		Limit range (%)	75.0	100.0	75.0	100.0
	Range lower than OPC	W/B ratio (%)	-	117.8–217.8	-	-
		Gel time (s)	-	20.0–50.0	-	-
Applicable range of W/B ratio shorten gel time than OPC (%)			-	117.8–177.1	-	-

**Table 7:** Estimated values of homogel strength according to W/B ratio (seven days).

Liquid B/CLGM (Vol.%)	Item		Estimated Values			
			CGM 1	CGM 2	CGM 3	OPC
50	Applicable range of W/B ratio	Maximum limit (%)	158.1	202.0	147.2	295.2
	Range higher than OPC	W/B ratio (%)	Below 135.2	Below 167.7	Below 124.1	-
		Homogel strength (MPa)	Higher than 4.8	Higher than 4.2	Higher than 5.0	-
60	Applicable range of W/B ratio	Maximum limit (%)	186.7	224.1	171.5	243.6
	Range higher than OPC	W/B ratio (%)	Below 172.1	Below 213.3	Below 156.5	-
		Homogel strength (MPa)	Higher than 4.0	Higher than 2.8	Higher than 4.4	-
70	Applicable range of W/B ratio	Maximum limit (%)	198.8	245.6	198.6	231.1
	Range higher than OPC	W/B ratio (%)	Below 187.0	Below 245.6	Below 187.6	-
		Homogel strength (MPa)	Higher than 3.7	Higher than 2.0	Higher than 3.6	-
Applicable range of W/B ratio Higher homogel strength than OPC (%)			Below 135.2	Below 167.7	Below 124.1	-

**Table 8:** Estimated values of gel time according to volume replacement ratio of Liquid B.

W/B (%)	Item		Estimated Values			
			CGM 1	CGM 2	CGM 3	OPC
100	Applicable range of Liquid B/CLGM	Maximum point (Vol.%)	35.6	-	23.1	20.0
		Minimum point (Vol.%)	64.1	56.7	69.2	62.9
		Limit range (Vol.%)	28.6	56.7	46.2	42.9
	Range lower than OPC	Liquid B/CLGM (Vol.%)	-	0.0–56.7	-	-
		Gel time (s)	-	20.0–50.0	-	-
120	Applicable range of Liquid B/CLGM	Maximum point (Vol.%)	39.3	8.0	25.3	26.7
		Minimum point (Vol.%)	72.6	68.0	85.3	69.5
		Limit range (Vol.%)	33.3	60.0	60.0	42.9
	Range lower than OPC	Liquid B/CLGM (Vol.%)	-	8.0–68.0	30.0–85.3	-
		Gel time (s)	-	20.0–50.0	20.0–47.7	-
140	Applicable range of Liquid B/CLGM	Maximum point (Vol.%)	47.4	30.0	48.6	34.4
		Minimum point (Vol.%)	80.7	72.9	91.4	80.5
		Limit range (Vol.%)	33.3	42.9	42.9	46.2
	Range lower than OPC	Liquid B/CLGM (Vol.%)	-	30.0–72.9	-	-
		Gel time (s)	-	20.0–50.0	-	-
Applicable range of Liquid B replacement ratio reduce gel time compared with that of OPC (Vol.%)			-	30.0–56.7	-	-

**Table 9:** Estimated values of homogel strength according to volume replacement ratio of Liquid B (7 days).

W/B (%)	Item		Estimated Values			
			CGM 1	CGM 2	CGM 3	OPC
100	Applicable range of Liquid B/CLGM	Minimum limit (Vol.%)	17.6	7.1	23.5	-
	Range higher than OPC	Liquid B/CLGM (Vol.%)	35.1–100.0	30.7–100.0	39.8–100.0	-
		Homogel strength (MPa)	4.2–27.3	3.9–21.2	4.6–29.0	-
120	Applicable range of Liquid B/CLGM	Minimum limit (Vol.%)	17.6	-	33.2	-
	Range higher than OPC	Liquid B/CLGM (Vol.%)	40.7–100.0	28.7–100.0	49.1–100.0	-
		Homogel strength (MPa)	4.5–21.1	3.8–17.0	4.9–27.1	-
140	Applicable range of Liquid B/CLGM	Minimum limit (Vol.%)	29.5	14.2	35.9	-
	Range higher than OPC	Liquid B/CLGM (Vol.%)	50.3–100.0	42.6–100.0	54.2–100.0	-
		Homogel strength (MPa)	4.7–20.6	4.4–17.3	4.8–21.7	-
Applicable range of Liquid B’s replacement ratio higher homogel strength than OPC (Vol.%)			50.3–100.0	42.6–100.0	54.2–100.0	-

## References

[1] Moseley M.P., Kirsch K. (2004). Ground Improvement.

[2] Akin M.K. (2016). Experimental studies on the physico-mechanical properties of jet-grout columns in sandy and silty soils. J. Afr. Earth Sci..

[3] Kazemian S., Prasad A., Huat B.B., Ghiasi V., Ghareh S. (2012). Effects of cement–sodium silicate system grout on tropical organic soils. Arab. J. Sci. Eng..

[4] Clarke W.J. (1984). Performance characteristics of microfine cement. Preprint.

[5] Karol R.H. (2013). Chemical Grouting and Soil Stabilization, Revised and Expanded.

[6] Shroff A.V., Shah D.L. (1999). Grouting Technology in Tunnelling and Dam Construction.

[7] Cristelo N., Soares E., Rosa I., Miranda T., Oliveira D.V., Silva R.A., Chaves A. (2013). Rheological properties of alkaline activated fly ash used in jet grouting applications. Constr. Build. Mater..

[8] Woyciechowski P., Woliński P., Adamczewski G. (2019). Prediction of carbonation progress in concrete containing calcareous fly ash co-binder. Materials.

[9] Escalante-Garcia J.I., Espinoza-Perez L.J., Gorokhovsky A., Gomez-Zamorano L.Y. (2009). Coarse blast furnace slag as a cementitious material, comparative study as a partial replacement of Portland cement and as an alkali activated cement. Constr. Build. Mater..

[10] Wang X.Y. (2019). Optimal design of the cement, fly ash, and slag mixture in ternary blended concrete based on gene expression programming and the genetic algorithm. Materials.

[11] Duxson P., Mallicoat S.W., Lukey G.C., Kriven W.M., van Deventer J.S.J. (2007). The effect of alkali and Si/Al ratio on the development of mechanical properties of metakaolin-based geopolymers. Coll. Surf. A. Physicochem. Eng. Asp..

[12] Juenger M.C.G., Winnefeld F., Provis J.L., Ideker J.H. (2011). Advances in alternative cementitious binders. Cem. Concr. Res..

[13] Duxson P., Fernández-Jiménez A., Provis J.L., Lukey G.C., Palomo A., van Deventer J.S.J. (2007). Geopolymer technology: The current state of the art. J. Mater. Sci..

[14] Kouamo H.T., Mbey J.A., Elimbi A., Kenne Diffo B.B., Njopwouo D. (2013). Synthesis of volcanic ash-based geopolymer mortars by fusion method: Effects of adding metakaolin to fused volcanic ash. Ceram. Int..

[15] Ul Haq E., Padmanabhan S.K., Licciulli A. (2014). Synthesis and characteristics of fly ash and bottom ash based geopolymers—A comparative study. Ceram. Int..

[16] Češnovar M., Traven K., Horvat B., Ducman V. (2019). The potential of ladle slag and electric arc furnace slag use in synthesizing alkali activated materials; the influence of curing on mechanical properties. Materials.

[17] Neville A.M. (1995). Properties of Concrete.

[18] Puertas F., Fernández-Jiménez A. (2003). Mineralogical and microstructural characterisation of alkali-activated fly ash/slag pastes. Cem. Concr. Comp..

[19] Bílek V. (2010). Preparation and stability of alkali activated materials from slag and fly ashes. Advances in Science and Technology.

[20] El-didamony H., Amer A.A., Ela-ziz H.A. (2012). Properties and durability of alkali-activated slag pastes immersed in sea water. Ceram. Int..

[21] Komljenović M., Baščarević Z., Marjanović N., Nikolić V. (2012). Decalcification resistance of alkali-activated slag. J. Hazard. Mater..

[22] Komljenović M., Baščarević Z., Marjanović N., Nikolić V. (2013). External sulfate attack on alkali-activated slag. Constr. Build. Mater..

[23] Panias D., Giannopoulou I.P., Perraki T. (2007). Effect of synthesis parameters on the mechanical properties of fly ash-based geopolymers. Coll. Surf. A. Physicochem. Eng. Asp..

[24] Wang M.R., Jia D.C., He P.G., Zhou Y. (2011). Microstructural and mechanical characterization of fly ash cenosphere/metakaolin-based geopolymeric composites. Ceram. Int..

[25] Baščarević Z., Komljenović M., Miladinović Z., Nikolić V., Marjanović N., Žujović Z., Petrović R. (2013). Effects of the concentrated NH_4_NO_3_ solution on mechanical properties and structure of the fly ash based geopolymers. Constr. Build. Mater..

[26] Cristelo N., Glendinning S., Teixeira Pinto A. (2011). Deep soft soil improvement by alkaline activation. Proc. Inst. Civil Eng. Ground Improv..

[27] Pacheco-Torgal F., Abdollahnejad Z., Camões A.F., Jamshidi M., Ding Y. (2012). Durability of alkali-activated binders: A clear advantage over Portland cement or an unproven issue?. Constr. Build. Mater..

[28] Li C., Sun H., Li L. (2010). A review: The comparison between alkali-activated slag (Si^+^ Ca) and metakaolin (Si^+^ Al) cements. Cem. Concr. Res..

[29] Gao X., Yu Q.L., Brouwers H.J.H. (2015). Properties of alkali activated slag–fly ash blends with limestone addition. Cem. Concr. Compos..

[30] Tomlinson M.J., Boorman R. (2001). Foundation Design and Construction.

[31] Anthony E.J., Granatstein D.L. (2001). Sulfation phenomena in fluidized bed combustion systems. Prog. Energy Combust. Sci..

[32] Koornneef J., Junginger M., Faaij A. (2007). Development of fluidized bed combustion—An overview of trends, performance and cost. Prog. Energy Combust. Sci..

[33] Armesto L., Merino J.L. (1999). Characterization of some coal combustion solid residues. Fuel.

[34] Lin W.T., Weng T.L., Cheng A., Chao S.J., Hsu H.M. (2018). Properties of controlled low strength material with circulating fluidized bed combustion ash and recycled aggregates. Materials.

[35] Mun K.J., Lee M.H., Yoon S.J. (2014). Development of non-cement material using recycled resources. Proc. Korea Inst. Build. Constr..

[36] Lunardi P. (1997). Ground improvement by means of jet-grouting. Proc. Inst. Civil Eng. Ground Improv..

[37] Kashevarova G., Makovetsky O., Khusainov I. (2013). Experience in application of “JET Grouting” for installation of substructures of estates. Front. Geotech. Eng..

[38] Akan R., Keskin S.N., Uzundurukan S. (2015). Multiple regression model for the prediction of unconfined compressive strength of jet grout columns. Proc. Earth Planet. Sci..

[39] Sun J. (2007). Project Specification of Grouting Method.

[40] American Society for Testing and Materials (1999). ASTM C109/C109M Standard Test Method for Compressive Strength of Hydraulic Cement Mortars.

